# Uncovering Suitable Reference Proteins for Expression Studies in Human Adipose Tissue with Relevance to Obesity

**DOI:** 10.1371/journal.pone.0030326

**Published:** 2012-01-17

**Authors:** Rafael Pérez-Pérez, Juan A. López, Eva García-Santos, Emilio Camafeita, María Gómez-Serrano, Francisco J. Ortega-Delgado, Wifredo Ricart, José M. Fernández-Real, Belén Peral

**Affiliations:** 1 Instituto de Investigaciones Biomédicas, Alberto Sols, Consejo Superior de Investigaciones Científicas (CSIC) & Universidad Autónoma de Madrid (UAM), Madrid, Spain; 2 CIBER Fisiopatología de la Obesidad y Nutrición (CIBERobn), ISCIII, Madrid, Spain; 3 Unidad de Proteómica, Centro Nacional de Investigaciones Cardiovasculares (CNIC), Madrid, Spain; 4 Department of Diabetes, Endocrinology and Nutrition, Hospital Dr. Josep Trueta, Girona, Spain; University of Tor Vergata, Italy

## Abstract

**Background:**

Protein expression studies based on the two major intra-abdominal human fat depots, the subcutaneous and the omental fat, can shed light into the mechanisms involved in obesity and its co-morbidities. Here we address, for the first time, the identification and validation of reference proteins for data standardization, which are essential for accurate comparison of protein levels in expression studies based on fat from obese and non-obese individuals.

**Methodology and Findings:**

To uncover adipose tissue proteins equally expressed either in omental and subcutaneous fat depots (study 1) or in omental fat from non-obese and obese individuals (study 2), we have reanalyzed our previously published data based on two-dimensional fluorescence difference gel electrophoresis. Twenty-four proteins (12 in study 1 and 12 in study 2) with similar expression levels in all conditions tested were selected and identified by mass spectrometry. Immunoblotting analysis was used to confirm in adipose tissue the expression pattern of the potential reference proteins and three proteins were validated: PARK7, ENOA and FAA. Western Blot analysis was also used to test customary loading control proteins. ENOA, PARK7 and the customary loading control protein Beta-actin showed steady expression profiles in fat from non-obese and obese individuals, whilst FAA maintained steady expression levels across paired omental and subcutaneous fat samples.

**Conclusions:**

ENOA, PARK7 and Beta-actin are proper reference standards in obesity studies based on omental fat, whilst FAA is the best loading control for the comparative analysis of omental and subcutaneous adipose tissues either in obese and non-obese subjects. Neither customary loading control proteins GAPDH and TBB5 nor CALX are adequate standards in differential expression studies on adipose tissue. The use of the proposed reference proteins will facilitate the adequate analysis of proteins differentially expressed in the context of obesity, an aim difficult to achieve before this study.

## Introduction

Quantitative proteomic and genomic technologies have recently revolutionized the search for disease-specific biomarkers or molecular signatures, enabling a better understanding of molecular events associated with disease development and progression, and eventually proposing therapeutic targets. At the protein expression level, confirmation of differences detected by proteomics is usually carried out by complementary approaches like immunoblotting analysis. Although classical Western Blot is typically used for qualitative purposes, quantification and accurate comparison of protein levels is feasible by the inclusion of reference standard proteins to better assess biological and experimental variations.

Ideally, a “good” internal reference control is expected to show constant expression levels across samples within a given experimental design. There is mounting evidence that housekeeping proteins used as ubiquitous loading controls, such as glyceraldehyde-3-phosphate dehydrogenase (GAPDH), Beta-actin and tubulin beta chain (TBB5) often show varying expression levels across samples and experimental conditions [1], [2], [3]. Since these proteins are also used in human adipose tissue expression studies, there are unmet needs to validate and/or identify a set of stable reference proteins for data standardization in this tissue.

Adipose tissue, previously seen as an inert fat depot, is now recognized as a highly active organ with many important physiological and pathological roles. This endocrine organ cross-talks with other essential organs acting as a crucial regulator of whole-body homeostasis [4], [5]. Human adipose tissue comprises two major intra-abdominal depots, the subcutaneous and the omental fat depot. A number of epidemiologic studies have shown that the size of the omental, more than the subcutaneous, fat is closely associated with a higher risk of obesity-related co-morbidities, such as insulin resistance, type 2 diabetes and cardiovascular disease [5], [6]. However, the underlying mechanisms for this association are not yet clear. Neither are the molecular mechanisms that lead to and take part in the development of obesity and the pathologies associated to obesity well understood.

Expression studies in adipose tissue are pivotal to gain insight into the mechanisms involved in obesity and its co-morbidities, and ultimately find novel markers and therapeutic targets. Recently a number of genomic and proteomic studies have revealed potential genes and proteins involved in obesity and/or type 2 diabetes [7], [8], [9], as well as differentially expressed genes and proteins in the omental and subcutaneous fat comparisons [10], [11], [12], [13], [14], [15]. Most studies resort to real time-PCR and Western Blot to validate these genes and proteins, respectively; however, despite that several genes have been proposed as suitable endogenous controls in gene expression studies on adipose tissue [16], [17], [18] no studies have attempted the identification and validation of adequate protein reference standards. As a consequence, expression studies continue to rely on customary loading controls (GAPDH, Beta-actin, TBB5) with unproven performance, which could overshadow the study conclusions owing to misinterpretation of results.

In the quest for reliable protein markers (i.e. showing steady expression) in human adipose tissue we have reanalyzed previously data from two-dimensional fluorescence difference gel electrophoresis (2D-DIGE) [10] and [19] for later protein identification by matrix-assisted laser desorption/ionization mass spectrometry (MALDI-MS) and Western Blot. Moreover we have studied, for the first time to our knowledge, reference standards frequently used in protein expression studies related to obesity research to find out their suitability as loading controls in immunoblotting analysis in human adipose tissue.

## Methods

### Ethics Statement

This study has been conducted according to the recommendations of the Declaration of Helsinki and was approved by the Ethics Committees of Hospital Dr. Josep Trueta (Girona, Spain). Signed informed consent was obtained from all subjects.

### Biological samples

Omental and subcutaneous adipose tissue samples used in 2D-DIGE analysis had been previously described [10] and [19]. Additional samples of omental and subcutaneous adipose tissue were obtained from 20 men and 30 women, who had been submitted for elective surgical procedures (cholecystectomy, surgery of abdominal hernia and gastric by-pass surgery). Clinical and biochemical characteristics of these 50 subjects are described in Table S1. Non-obese subjects had a body mass index (BMI)<30 kg/m^2^ and obese subjects had a BMI>30 kg/m^2^. During surgery, biopsies of adipose tissues were obtained after an overnight fast, washed in chilled 9 g/L NaCl solution, partitioned into pieces, and immediately frozen in liquid nitrogen and stored at −80°C until protein extraction. The surgeon aimed to obtain the samples from similar anatomical locations in all the subjects. All subjects were of Caucasian origin and reported that their body weight had been stable for at least three months before the study. None of the subjects had type 2 diabetes or any other systemic disease apart from obesity and all were free of any infections within the previous month before the study. Liver disease and thyroid dysfunction were specifically excluded by biochemical work-up. Other exclusion criteria for those patients included the following: 1) clinically significant hepatic, neurological, or other major systemic disease, including malignancy; 2) history of drug or alcohol abuse, defined as >80 g/day, or serum transaminase activity more than twice the upper normal range limit; 3) elevated serum creatinine concentrations; 4) acute major cardiovascular event in the previous 6 months; 5) acute illnesses and current evidence of chronic inflammatory or infectious diseases; and 6) mental illness rendering the subjects unable to understand the nature, scope, and possible consequences of the analysis.

### 2D-DIGE separation and analysis

Protein extract preparation and separation by 2D-DIGE have been independently described for study 1, which compared omental and subcutaneous fat depots from obese individuals [10] and for study 2, which compared omental adipose tissue from non-obese and obese individuals [19]. Briefly, proteins were extracted from adipose tissue biopsies and the concentration was determined using the RC/DC Protein Assay (Bio-Rad Laboratories, Hercules, CA, USA). Protein extracts were labelled with Cy2, Cy3 or Cy5 fluorescent cyanine dyes, and were mixed and separated on 2-DE gels using 24 cm pH 3–11 NL strips in the first dimension and 12% SDS-PAGE gels in the second dimension. After SDS-PAGE, gels were scanned with a Typhoon 9400 fluorescent scanner (GE Healthcare) at 100 µm resolution using appropriate individual excitation and emission wavelengths and filters. Gel images previously analyzed with the DeCyder software (GE Healthcare) to detect the corresponding proteins differentially expressed in either study 1 [10] and study 2 [19] were now reanalyzed with DeCyder software v7 to reveal protein spots which remained significantly unaffected (i.e. with average ratio around 1) across samples in each study.

### In-gel trypsin digestion and mass spectrometry

Protein spots which remained significantly unaffected across samples as revealed by DeCyder software were selected for gel excision from silver-stained gels, digested automatically and analyzed in an Ultraflex MALDI TOF/TOF mass spectrometer (Bruker Daltonik, Bremen, Germany) [20] to obtain the corresponding MALDI-MS and MALDI-MS/MS spectra. In a first step, MALDI-MS spectra were acquired by averaging 300 individual spectra in the positive ion reflector mode at 50 Hz laser frequency in a mass range from 800 to 4000 Da. In a second step, precursor ions showing in the MALDI-MS mass spectrum were subjected to fragment ion analysis in the tandem (MS/MS) mode to average 1000 spectra. Peak labeling, internal calibration based on two trypsin autolysis ions with m/z = 842.510 and m/z = 2211.105, as well as removal of known trypsin and keratin peptide masses were performed automatically using the flexAnalysis 2.2 software (Bruker Daltonik). No smoothing or any further spectral processing was applied. MALDI-MS and MS/MS spectra were manually inspected in detail and reacquired, recalibrated and/or relabeled using the aforementioned programs and homemade software when necessary.

### Database searching

MALDI-MS and MS/MS data were combined through the BioTools 3.0 program (Bruker Daltonik) to search a nonredundant protein database (NCBInr 20091022; ∼7.0×10^6^ entries; National Centre for Biotechnology Information, Bethesda, US), using the Mascot 2.2 software (Matrix Science, London, UK; http://www.matrixscience.com) [21]. Other relevant search parameters were set as follows: enzyme, trypsin; fixed modifications, carbamidomethyl (C); allow up to 1 missed cleavage; peptide tolerance ±20 ppm; MS/MS tolerance ±0.5 Da. Protein scores greater than 81 were considered significant (p<0.05).

### Immunoblotting analysis

Fat tissue was homogenized in radioimmnuno precipitation assay (RIPA) buffer as described in [10]. Protein extracts (10 µg) were loaded, resolved on 10–14% SDS-PAGE and transferred to Hybond ECL nitrocellulose membranes by conventional procedures. Membranes were stained with 0.15% Ponceau red (Sigma-Aldrich, St Louis, MO, USA) to ensure equal loading after transfer and then blocked with 5% (w/v) BSA or dried nonfat milk in TBS buffer with 0.1% Tween 20. The antibodies used for Western Blot analysis revealed in each case single bands at the expected molecular masses. The primary antibodies used were 1∶10000 rabbit anti-ENOA (sc-15343), 1∶200 goat anti-PARK7 (sc-27004), 1∶1000 goat anti-FAA (sc-66223), 1∶2000 goat anti-Beta-actin (sc-1616), 1∶500 goat anti-PGK1 (sc-17943), 1∶5000 mouse anti-TBB5 (sc-134234), and 1∶5000 rabbit anti-CALX (sc-11397) all from Santa Cruz Biotechnology, Inc (Heidelberg, Germany) and 1∶4000 mouse anti-GAPDH, AM4300 (Ambion, Austin, USA). Blots were incubated with the appropriate IgG-HRP-conjugated secondary antibody. Immunoreactive bands were visualized with ECL-plus reagent kit (GE Healthcare). Blots were exposed for different times; exposures in the linear range of signal were selected for densitometric evaluation. Optical densities of the immunoreactive bands were measured using Image J analysis software.

### Immunohistochemistry

Five-micron sections of formalin-fixed paraffin-embedded adipose tissue were deparaffinized and rehydrated prior to antigen unmasking by boiling in 1 mM EDTA, pH 8. Sections were blocked in normal serum and incubated overnight with the following antibodies: rabbit anti-ENOA (sc-15343) at 1∶100 dilution, goat anti-PARK7 (sc-27004) at 1∶50 dilution, and goat anti-FAA (sc-66223) at 1∶50 dilution. Secondary antibody staining was performed using the VECTASTAIN ABC kit (Vector laboratories, Inc Burlingame, CA) and detected with diaminobenzidine (DAB, Vector Laboratories, Inc). Sections were counterstained with hematoxylin prior to dehydration and coverslip placement. As a negative control, the entire immunohistochemical procedure was performed on adjacent sections in the absence of primary antibody.

### Statistical analyses

Descriptive results of continuous variables are expressed as mean ± standard deviation (SD). Before statistical analysis, normal distribution and homogeneity of the variances were evaluated using *Levene's* test. One-way ANOVA, for multiple comparisons, using post-hoc by *Bonferroni's* test (when equal variances could be assumed), was used to compare groups. The statistical analyses were performed using the program SPSS 16.0 (SPSS Inc., Illinois, USA). Statistical comparisons of the Western Blot densitometry data were carried out using the Student's *t* test for paired (omental *vs*. subcutaneous) and independent (obese *vs*. non-obese) samples, and results were expressed as means ± SD using SPSS. The coefficient of variation (CV) was also calculated. Statistical significance was set at p<0.05.

## Results

### Reference proteins for comparative expression studies between omental and subcutaneous adipose tissue

**Figure 1:**
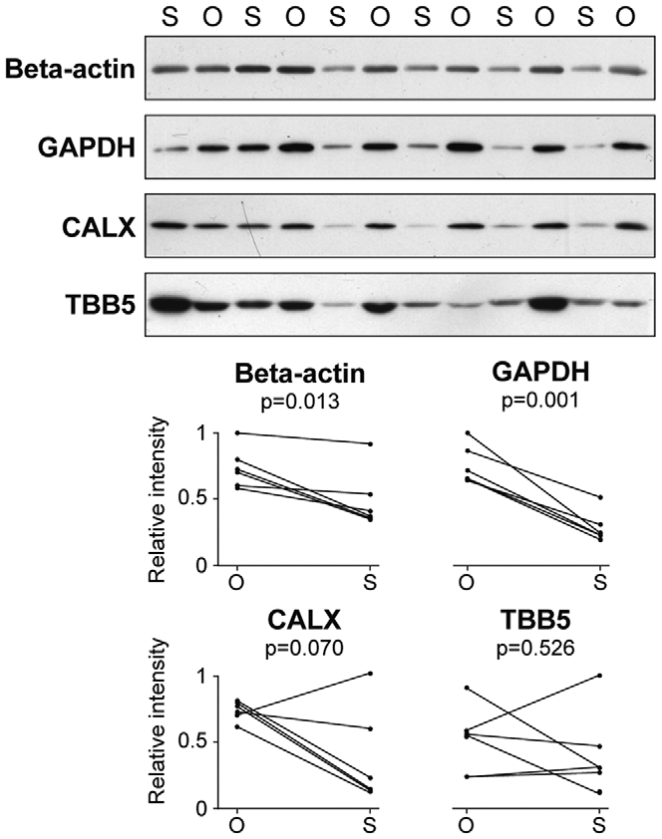
Protein expression levels in human omental and subcutaneous adipose tissue. Representative Western Blot analysis of Beta-actin, GAPDH, CALX and TBB5 in omental (O) and subcutaneous (S) fat samples. Paired relative intensity values from band densitometry in omental and subcutaneous fat samples from the same individual are represented.

We had previously found over-expression of GAPDH in the omental compared to the subcutaneous human adipose tissue [10]. Given that this protein is a customary loading control in expression studies, we evaluated other common reference proteins, namely Beta-actin and TBB5, as well as calnexin (CALX) in omental and subcutaneous adipose tissue by Western Blot. We have tested samples from 50 patients, including males and females across a wide BMI range and a wide age range, as described in Table S1. Beta-actin and CALX levels were higher in the omental than in the subcutaneous fat (p<0.05), as was the case for GAPDH, while TBB5 showed a very heterogeneous expression profile across samples (coefficient of variation, CV = 59%). Figure 1 shows a representative group of samples to illustrate the expression pattern of these proteins. Results demonstrate that neither customary Beta-actin, TBB5 and GAPDH nor CALX are adequate reference standards for protein expression studies addressing the comparison of omental and subcutaneous samples.

**Figure 2:**
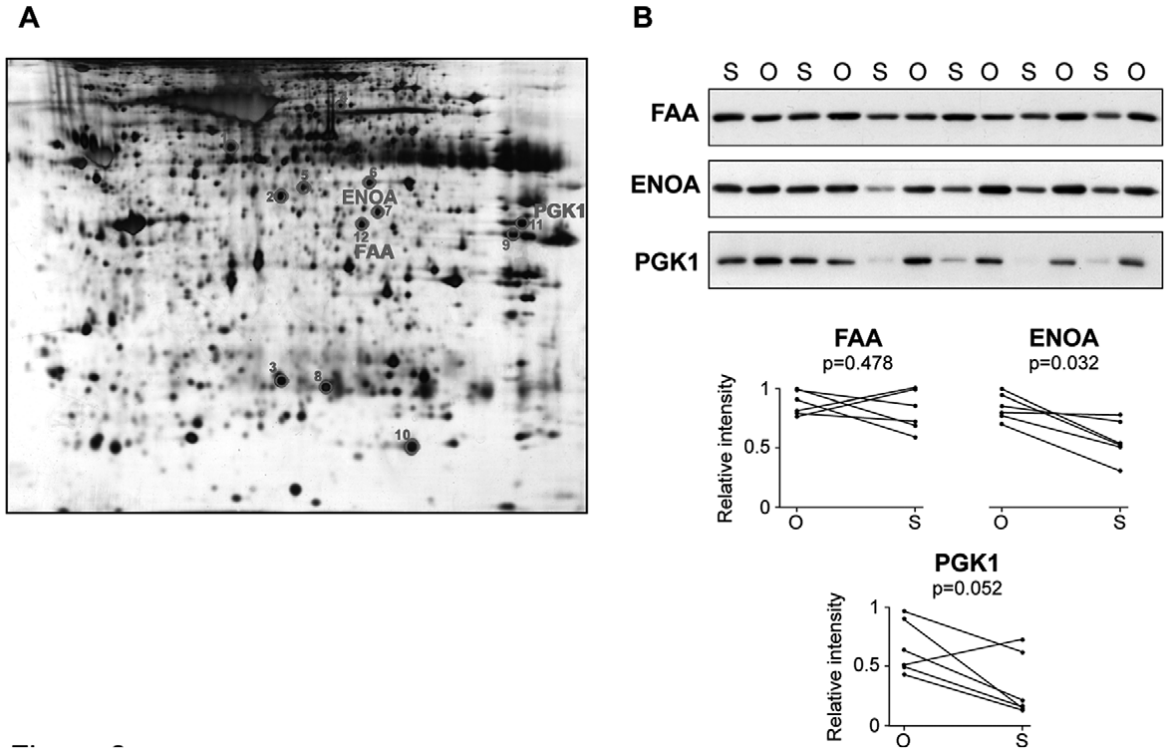
Proteins identified by 2D-DIGE and MS showing sustained expression levels in omental and subcutaneous adipose tissues. Protein extracts from subcutaneous and omental adipose tissue paired samples were analyzed by 2D-DIGE [10]. Proteins were revealed by AgNO_3_ staining and based on DeCyder analysis the spots of interest were excised from the gel and subject to MS-based identification. A representative silver-stained gel is shown; the numbers correspond to 12 selected protein spots as indicated in Table 1 (A). Representative Western Blot analysis of selected proteins in omental (O) and subcutaneous (S) fat samples. Paired relative intensity values from band densitometry in fat samples from the same individual are represented (B).

In view of the lack of suitable standard proteins, a reanalysis was accomplished with data from our previously published proteomic study based on 2D-DIGE and MALDI-MS analyses [10] to uncover appropriate reference standards. Twelve spots with similar expression levels across samples were selected and identified by MALDI-MS (Table 1 and Figure 2A).

**Table 1:** Candidate reference proteins identified by MALDI-MS study.

Spot^a^	Accesion Code NCBI	Accession Code Swiss-Prot	Protein Description	Mascot Score	Mascot Expect	Mascot Ions score	MW (kDa)	pI	Matched/Unmatched peptides	Coverage
Omental *vs*. subcutaneous										
1	gi|119597640	PDIA3_HUMAN	Protein disulfide isomerase family A, member 3, isoform CRA_a	278	1.30E-21	95	54.4	6.78	13/3	31
2	gi|18379349	VAT1_HUMAN	Vesicle amine transport protein 1	220	8.00E-16	88	42.1	5.88	7/1	27
3	gi|1922287	ECHS1_HUMAN	Enoyl-CoA hidratase, mitochondrial	234	3.20E-17	103	31.8	8.34	8/2	26
4	gi|3420181	WDR1_HUMAN	WDR1 protein	106	0.0002	74	58.6	6.41	2/0	4
5	gi|169646441	GDI2_HUMAN	GDP dissociation inhibitor 2 isoform 2	184	3.20E-12	80	46.0	5.91	7/1	25
6	gi|39644728	ENOA_HUMAN	Enolase 1	143	4.60E-08	80	29.2	5.87	6/1	19
7	gi|3641398	IDH1_HUMAN	NADP-dependent isocitrate dehydrogenase	214	3.20E-15	75	46.9	6.34	10/2	23
8	gi|4758638	PRDX6_HUMAN	Peroxiredoxin 6	191	6.30E-13		25.1	6	10/1	39
9	gi|119587507	THIL_HUMAN	Acetyl-CoA acetyltransferase, mitochondrial	96	0.002	74	21.6	9.28	1/0	6
10	gi|4503057	CRYAB_HUMAN	Crystallin, alpha B	134	3.20E-07	89	20.1	6.76	7/1	22
11	gi|4505763	PGK1_HUMAN	Phosphoglycerate kinase 1	194	8.90E-15	120	45.0	8.3	5/0	18
12	gi|4557587	FAA_HUMAN	Fumarylacetoacetase	98	0.0018	79	46.7	6.46	2/0	3
Obese *vs*. non-obese										
13	gi|203282367	ENOA_HUMAN	Enolase 1	298	1.60E-23		47.3	6.99	19/3	45
14	gi|5453597	CAZA1_HUMAN	F-actin capping protein alpha-1 subunit	203	5.00E-14	103	33.1	5.45	5/1	29
15	gi|4504981	LEG1_HUMAN	Galectin-1	212	1.40E-16	155	15.0	5.34	3/0	29
16	gi|6598323	GDIB_HUMAN	GDP dissociation inhibitor 2 isoform 1	410	9.90E-35	135	51.1	6.11	18/4	45
17	gi|4557587	FAA_HUMAN	Fumarylacetoacetase	177	2.00E-11	85	46.7	6.46	6/1	14
18	gi|9951915	SAHH_HUMAN	Adenosylhomocysteinase isoform 1	183	5.00E-12	90	48.2	5.92	5/1	14
19	gi|4503481	EF1G_HUMAN	Eukaryotic translation elongation factor 1 gamma	363	5.00E-30	165	50.4	6.25	16/2	37
20	gi|25777615	PSD7_HUMAN	Proteasome 26S non-ATPase subunit 7	145	7.10E-10	59	37.1	6.29	4/1	21
21	gi|31543380	PARK7_HUMAN	Parkinson disease protein 7	241	1.80E-19	139	20.0	6.33	5/2	26
22	gi|5803187	TALDO_HUMAN	Transaldolase 1	104	8.90E-06	48	37.7	6.36	3/1	10
23	gi|119619009	PGK1_HUMAN	Phosphoglycerate kinase 1, isoform CRA_b	291	7.40E-23	194	28.0	9.23	5/2	23
24	gi|4503571	ENOA_HUMAN	Enolase 1	182	6,30E-12		47.5	7,01	10/2	32

Protein spots with similar expression levels across samples were identified by MALDI-MS after comparative analyses of omental *vs.* subcutaneous adipose tissue (spots from 1 to 12) and omental fat from obese *vs*. non-obese subjects (spots from 13 to 24).

a Spot numbering as in Figure 2A (1–12) and Figure 4A (13–24).

Based on 2D-DIGE results, a set of three least-variable proteins, namely enolase 1 (ENOA), phosphoglycerate kinase 1 (PGK1) and fumarylacetoacetase (FAA), were evaluated by Western Blot in fat tissue samples from the subjects included in the study. Figure 2B shows expression levels of these three proteins in a representative group of samples. Results demonstrate that FAA maintained steady expression levels across paired omental and subcutaneous fat samples, with a CV of 16%, ENOA was over-expressed in the omental fat biopsies compared to subcutaneous fat, and PGK1 showed variable expression levels across the majority of paired samples, presenting a CV of 59% (Figure 2B). Consequently, FAA was the most suitable loading control for studying differential expression between omental and subcutaneous adipose tissue.

### Reference proteins for comparative expression studies in omental adipose tissue from obese and non-obese individuals

**Figure 3:**
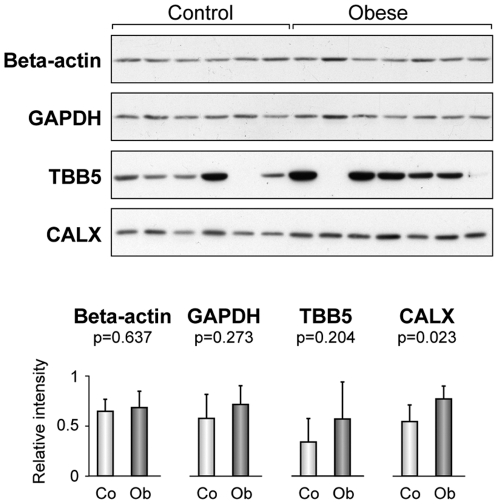
Protein expression levels in omental adipose tissue from non-obese and obese subjects. Representative Western Blot analysis of Beta-actin, GAPDH, TBB5 and CALX expression in omental fat samples from non-obese (control) and obese subjects. Relative intensity values from band densitometry are expressed as mean ± SD.

The expression patterns of GAPDH, Beta-actin, TBB5 and CALX were evaluated by immunoblotting analysis, using omental fat protein extracts from non-obese and obese patients. Expression levels of GAPDH, TBB5 and CALX were higher in obese patients. GAPDH and TBB5 showed considerable intra-group variation, especially TBB5 with CV = 70% (Figure 3). Accordingly, these proteins do not constitute appropriate loading controls in comparative protein expression studies comprising non-obese and obese individuals. On the contrary, results unveiled that Beta-actin was an appropriate reference standard, albeit the moderate intra-group variation observed for this protein (CV = 21%).

**Figure 4:**
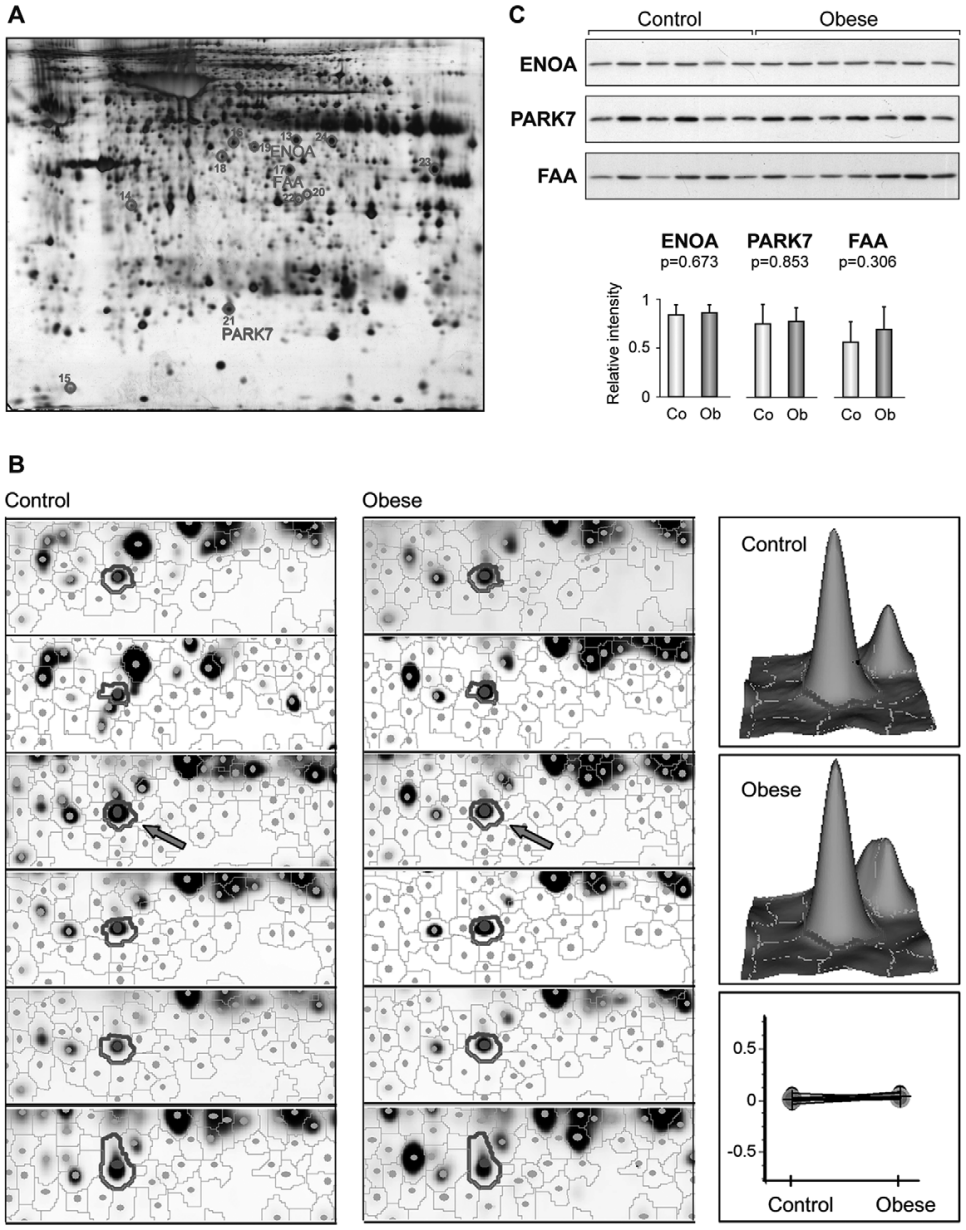
Proteins identified by 2D-DIGE and MS showing sustained expression levels in omental adipose tissue from non-obese and obese subjects. Protein extracts from omental fat of obese and non-obese subjects were analyzed by 2D-DIGE as described [19]. A selected silver-stained gel is shown with spots numbered as indicated in Table 1 (A). Sustained expression of PARK7 in obese and non-obese (control) subjects by DeCyder analysis. Zoomed images of 2D-DIGE gels around PARK7 spot (circled), 3D-view of selected spots (arrow) and graphical representation of the Standardized Abundance (Log) of PARK7 spot is shown (B). Representative Western Blot analysis of ENOA, PARK7 and FAA expression in omental fat samples from non-obese and obese subjects. Relative intensity values from band densitometry are expressed as mean ± SD (C).

To uncover alternative suitable reference proteins, a reanalysis was accomplished with data from our previous proteomic study comparing omental fat biopsies from six obese and six non-obese subjects based on 2D-DIGE [19]. Twelve protein spots with steady levels across samples were selected and excised from silver-stained gels, digested with trypsin, and analyzed by MALDI-MS (Table 1 and Figure 4A), including ENOA, FAA and Parkinson disease protein 7 (PARK7), which showed the least variability across samples according to DeCyder software. This is illustrated in Figure 4B, where representative zoomed images of 2D-DIGE gels around PARK7 spot demonstrate sustained levels of this protein, with a fold change ratio between the two sample groups of 1.07.

These three proteins were further evaluated by Western Blot using omental samples from non-obese and obese subjects. This study revealed nearly invariable ENOA and PARK7 levels in obese and non-obese individuals, together with a highly homogeneous intra-group expression pattern across samples (SD 0.11 and 0.13 for non-obese and obese samples, respectively, with ENOA; SD 0.2 and 0.14 with PARK7). CV values were 16% for ENOA and 22% for PARK7, the latter being similar to the CV value of Beta-actin. FAA exhibited slightly heterogeneous expression patterns across samples, with a CV of 35% (Figure 4C). Therefore, our studies unveiled that ENOA, PARK7 and Beta-actin can be considered adequate reference proteins for expression studies in human omental adipose tissue in non-obese and obese subjects.

To extend the validity of the proposed protein markers to tissues from obese type 2 diabetic patients, the expression levels of ENOA, PARK7, FAA, Beta-actin, GAPDH, TBB5 and CALX levels were compared by immunoblotting analysis. We used omental adipose tissue proteins from non-obese and non-diabetic subjects *vs*. obese and type 2 diabetic patients. Type 2 diabetes was diagnosed when fasting serum glucose was above 126 mg/dl or serum glucose 2 hour after oral glucose tolerance test was above 200 mg/dl, repeated at least 2 times. ENOA and PARK7 showed the most steady expression profiles across samples with CV of 13% and 20%, respectively, therefore revealing as the most appropriate reference standards when comparing non-diabetic and type 2 diabetic subjects (Figure S1).

## Discussion

Reference standards with steady expression across tissue sample cohorts are indispensable in clinical research to reliably determine altered proteins associated to human disease. We have successfully identified and validated proteins with sustained expression levels to be used as reference standards in expression studies on human adipose tissue with relevance to the pathophysiology of obesity. An objective of many differential expression studies is to define protein profiles that can distinguish between normal and pathological states, enabling a better understanding of molecular events associated with disease development and progression, and eventually proposing novel biomarkers or therapeutic targets. Confirmation of differences at the protein expression level is usually carried out by immunoblotting analysis, which becomes quantitative by using loading controls. Nevertheless, these quantitative measurements are prone to error due to overloading of internal standards and over-reliance on normalization [1], [3]. With the antibodies and experimental conditions used in this study, 10 µg of proteins have enabled linearity in Western Blot analysis. This emphasizes the need to optimize experimental conditions for a given assay, including avoidance of blot overexposure to ensure a linear relation between protein concentration and Western Blot signal.

Most reference controls consist of housekeeping genes and proteins, as these are constitutively expressed for functions required for cell maintenance. However, lately many gene expression studies, a few of them based on adipose tissue, have shown that several of the commonly used housekeeping genes may be unsuitable due to the influence of physiological and pathological events on their expression [17], [18]. Although a few protein studies have addressed this point [2], [22], no systematic work has evaluated customary reference proteins in adipose tissue, and therefore expression analyses continue to rely on Beta-actin, TBB5, GAPDH and/or CALX [23], [24], [25], [26], [27], [28], [29]. It is noteworthy that constitutive expression in cells does not preclude regulation; in fact our results show that most proteins currently used as loading controls cannot be regarded suitable reference standards in human adipose tissue. While Beta-actin is the only customary reliable reference when comparing omental fat from obese and non-obese subjects, it is utterly inadequate for the comparison of omental and subcutaneous fat. As a consequence, housekeeping proteins to be used as reference standards should be carefully assayed.

Consequently, we resorted for the first time to 2D-DIGE, MS and immunoblotting analyses to identify adipose tissue reference proteins equally expressed in the two major intra-abdominal fat depots, the omental and the subcutaneous depots, across a wide adiposity range. In addition, since omental adipose tissue has been linked to many of the morbidities associated with obesity, including type 2 diabetes and cardiovascular disease [5], [6], we have searched for reference proteins by comparing omental fat biopsies from obese and non-obese subjects. In fact, it has been speculated whether omental fat would be a causal factor, or a marker of “dysfunctional adipose tissue” [30].

**Figure 5:**
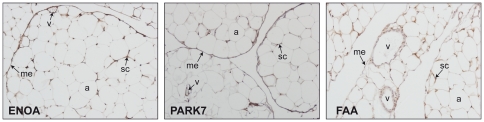
Immunohistochemical detection of ENOA, PARK7 and FAA in omental adipose tissue. Images are representative of adipose tissue sections collected from five subjects. Magnification ×100. **a**, adipocyte; **me**, mesothelium; **sc**, stromal cells; **v**, vessel.

Our study has revealed a trend to increased protein levels in the omental versus subcutaneous fat (Figure 1 and Figure 2B). Mesothelium tissue, which is present only in the omental fat [31], could in some instances account for these increased levels, as is the case for ENOA, which is also expressed in the mesothelial cells (Figure 5). Nevertheless, ENOA expression remained steady in the omental fat from non-obese and obese individuals. It is not surprising that this highly conserved, multifunctional enzyme involved in a variety of cellular biological processes such as growth control, may exert similar functions regardless of obesity and/or type 2 diabetes states. Likewise, PARK7 showed stable expression levels in non-obese and obese adipose tissue. The product of the gene *DJ-1* is a highly conserved protein implicated in a number of cell processes. First identified as an oncogene driving to abnormal cellular responses related to cell survival [32], *DJ-1* is also recognized as one of the defective genes in Parkinson disease, due to loss-of-function mutations [33]. Although PARK7 has been found modulated in several pathological conditions, such as endometriosis and hepatocellular carcinoma [34], [35], [36], our results, supported by two techniques, show that PARK7 is not differentially expressed in obesity. As far as we know, this protein had not been studied in human adipose tissue yet. Our findings based on immunohistochemical analyses have shown for the first time expression of PARK7 in adipocytes, mesothelial cells and other stromal cells in omental adipose tissue, supporting its ubiquitous expression [37]. PARK7 antibody stained mainly the nucleus of cells, but cytoplasm expression was also observed (Figure 5). It is noteworthy that the optional use of PARK7 or ENOA (19.9 and 47.2 kDa, respectively) enables quantitative Western Blot analysis in a wide range of protein molecular weight.

Conversely, FAA maintained steady expression levels across paired omental and subcutaneous fat samples. Neither had FAA been studied in adipose tissue yet; this protein acts as the terminal enzyme in the tyrosine catabolism pathway, and its defects cause an autosomal recessive inborn error of metabolism named Type 1 tyrosinemia (HT) which has been studied mostly in liver and kidney, two of the organs affected in this disorder [38]. To our knowledge no proteins with comparable expression levels between omental and subcutaneous adipose tissue have been reported yet. Immunohistochemical analysis using an FAA antibody provided further evidence, as mesothelial cells in omental adipose tissue fail to stain (Figure 5).

In summary, we have unveiled that neither customary GAPDH and TBB5 nor CALX can be regarded proper loading controls in differential protein expression studies on adipose tissue. Consequently, using proteomic approaches we have demonstrated that Beta-actin, ENOA and PARK7 are the most adequate reference standards in obesity studies based on human omental adipose tissue, whilst FAA is the best loading control for the comparative analysis of omental and subcutaneous fat depots. The use of the reference proteins proposed in this work will facilitate the adequate analysis of proteins differentially expressed in adipose tissue in the context of obesity, a goal difficult to meet before this study.

## Supporting Information

Figure S1 **Protein expression levels in omental adipose tissue from non-obese and non-diabetic, and type 2 diabetic obese subjects.** Representative Western Blot analysis of ENOA, PARK7, FAA, Beta-actin, GAPDH, TBB5 and CALX expression in omental fat samples from non-obese and non-diabetic (control), and type 2 diabetic obese subjects. ENOA, PARK7 and Beta-actin proteins show steady expression profiles across samples with CV of 13%, 20% and 28% repectively. Relative intensity values from band densitometry are expressed as mean ± SD.
(TIF)

Table S1 **Clinical characteristics of the individuals included in this study.**
(DOC)
