# Supplementary figures and images for: Uncovering Suitable Reference Proteins for Expression Studies in Human Adipose Tissue with Relevance to Obesity
Source: PLoS One. 2012 Jan 17;7(1):e30326. doi: 10.1371/journal.pone.0030326 (PMC3260266; doi:10.1371/journal.pone.0030326)

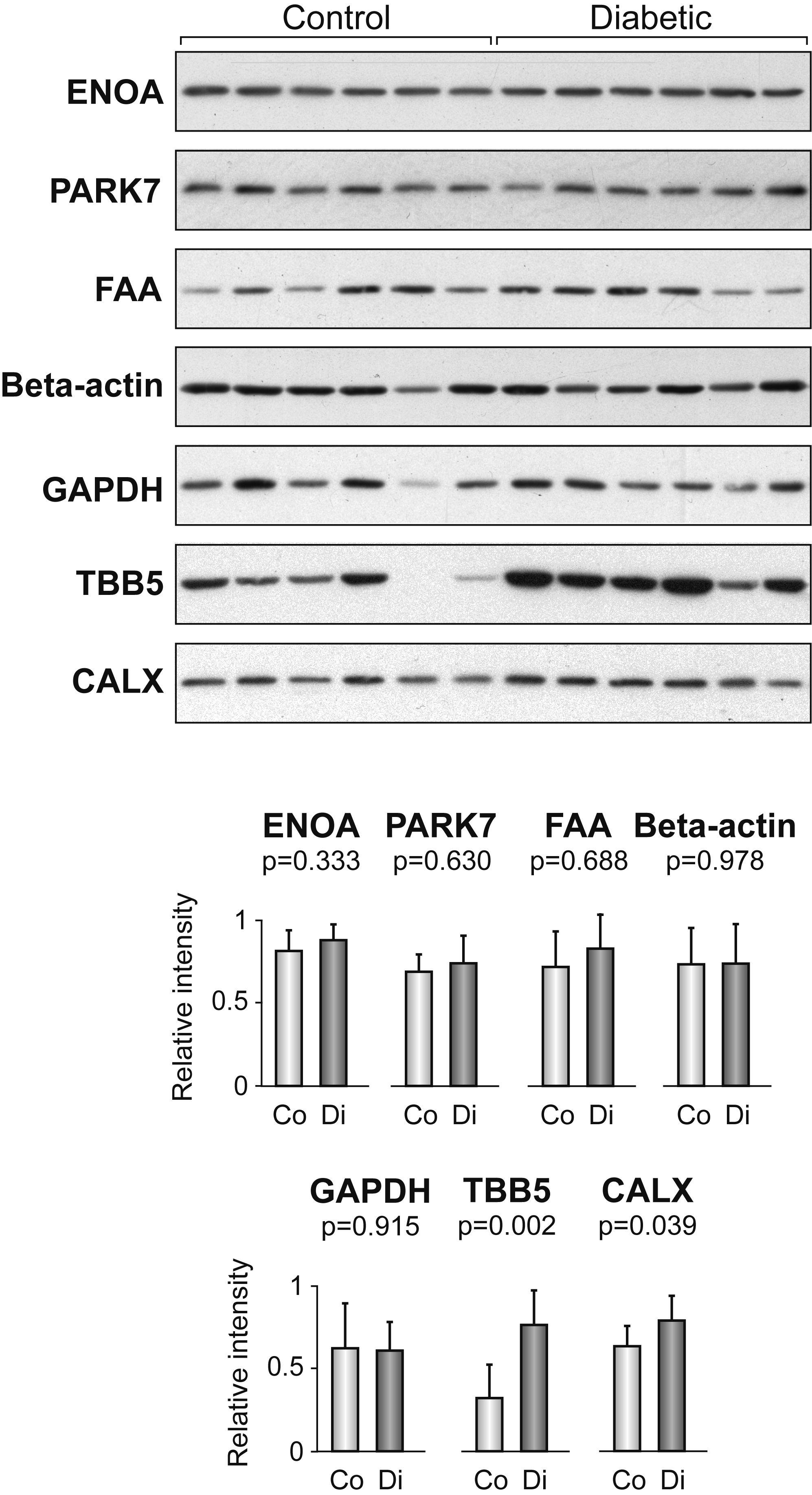

Supplement: Figure S1 — Protein expression levels in omental adipose tissue from non-obese and non-diabetic, and type 2 diabetic obese subjects. Representative Western Blot analysis of ENOA, PARK7, FAA, Beta-actin, GAPDH, TBB5 and CALX expression in omental fat samples from non-obese and non-diabetic (control), and type 2 diabetic obese subjects. ENOA, PARK7 and Beta-actin proteins show steady expression profiles across samples with CV of 13%, 20% and 28% repectively. Relative intensity values from band densitometry are expressed as mean ± SD. (TIF) [file pone.0030326.s001.tif]
