# Supplementary material for: Uncovering Suitable Reference Proteins for Expression Studies in Human Adipose Tissue with Relevance to Obesity
Source: PLoS One. 2012 Jan 17;7(1):e30326. doi: 10.1371/journal.pone.0030326 (PMC3260266; doi:10.1371/journal.pone.0030326)
Supplement: Table S1 — Clinical characteristics of the individuals included in this study. (DOC) [file pone.0030326.s002.doc]

**Table S1.** Clinical characteristics of the individuals included in this study

Clinical characteristics of females

|  | **Non-obese**  **(BMI<30)** | **Morbid obese (40<BMI)** | ***P (t-student)*** |
| --- | --- | --- | --- |
| **N (women)** | 15 | 15 |  |
| **Age (years)** | 45 ±10 | 48 ±6 | 0.455 |
| **BMI (kg/m^2^)** | **26.3 ±2.9** | **44.6 ±3.4** | ***<0.0001*** |
| **Fat mass (%)** | **36.6 ±4.4** | **59.1 ±4.6** | ***<0.0001*** |
| **Waist (cm)** | **87.1 ±5.7** | **118.5 ±12.0** | ***0.001*** |
| **Hip (cm)** | **96.2 ±5.9** | **151.0 ±19.8** | ***<0.0001*** |
| **Waist-to-Hip ratio** | **0.89 ±0.033** | **0.78 ±0.02** | ***0.007*** |
| **SBP (mmHg)** | **126.8 ±14.8** | **145.8 ±9.9** | ***0.030*** |
| **DBP (mmHg)** | **73.5 ±10.9** | **87.5 ±8.7** | ***0.035*** |
| **Fasting glucose (mg/dL)** | 86.3 ±12.6 | 97.1 ±14.4 | 0.075 |
| **HbA_1c_ (%)** | 4.7 ±0.7 | 4.8 ±0.3 | 0.697 |
| **Total cholesterol (mg/dL)** | 200.1 ±35.3 | 202.9 ±29.8 | 0.856 |
| **HDL-cholesterol (mg/dL)** | 75.5 ±27.3 | 57.5 ±16.4 | 0.111 |
| **LDL-cholesterol (mg/dL)** | 101.7 ±41.8 | 115.7 ±15.3 | 0.318 |
| **Fasting Triglycerides (mg/dL)** | 118.4 ±67.2 | 125.9 ±53.0 | 0.797 |

Clinical characteristics of males

|  | **Non-obese**  **(BMI<30)** | **Obese**  **(30≤BMI<40)** | **Morbid obese (40≤BMI)** | ***P (ANOVA)*** |
| --- | --- | --- | --- | --- |
| **N (men)** | 6 | 4 | 10 |  |
| **Age (years)** | 50 ±13 | 43 ±5 | 41 ±9 | 0.177 |
| **BMI (kg/m^2^)** | **26.6 ±2.2** | **35.6** ±3.4 | **49.5 ±3.9** | ***<0.0001*** |
| **Fat mass (%)** | **27.5 ±2.0** | **40.9** ±8.5 | **50.7 ±5.4** | ***<0.0001*** |
| **SBP (mmHg)** | 140.2 ±13.4 | 134.5 ±31.8 | 132.3 ±10.1 | 0.799 |
| **DBP (mmHg)** | 83.0 ±5.1 | 88.5 ±14.8 | 84.0 ±6.6 | 0.702 |
| **Fasting glucose (mg/dL)** | 84.7 ±7.0 | 92.5 ±17.9 | 115.4 ±60.5 | 0.394 |
| **HbA_1c_ (%)** | 5.0 ±0.3 | 4.6 ±0.4 | 4.9 ±0.6 | 0.506 |
| **Total cholesterol (mg/dL)** | 203.8 ±55.1 | 168.3 ±14.0 | 174.9 ±35.3 | 0.397 |
| **HDL-cholesterol (mg/dL)** | 47.9 ±15.6 | 68.3 ±32.1 | 41.6 ±11.8 | 0.091 |
| **LDL-cholesterol (mg/dL)** | 122.1 ±28.1 | 76.3 ±34.3 | 100.6 ±18.4 | 0.076 |
| **Fasting Triglycerides (mg/dL)** | 139.0 ±77.2 | 108.3 ±76.2 | 143.6 ±97.5 | 0.840 |

Data are means ±SD; **BMI,** Body Mass Index; **SBP,** systolic blood pressure; **DBP,** diastolic blood pressure; **HbA_1c_,** glycated hemoglobin.
